# Altitudinal variations in wing morphology of *Aedes albopictus* (Diptera, Culicidae) in Albania, the region where it was first recorded in Europe

**DOI:** 10.1051/parasite/2019053

**Published:** 2019-09-06

**Authors:** Jorian Prudhomme, Enkelejda Velo, Silvia Bino, Perparim Kadriaj, Kujtim Mersini, Filiz Gunay, Bulent Alten

**Affiliations:** 1 UMR MIVEGEC (IRD 224 – CNRS 5290 – Université de Montpellier) 911 Avenue Agropolis 34394 Montpellier France; 2 Department of Control of Infectious Diseases, Institute of Public Health Str. “Aleksandër Moisiu” No. 80 1010 Tirana Albania; 3 Southeast European Center for Surveillance and Control of Infectious Diseases (SECID) Str. “Aleksandër Moisiu” No. 80 1010 Tirana Albania; 4 Faculty of Science, Department of Biology, Ecology Section, Vector Ecology Research Group Laboratories, Hacettepe University 06800 Ankara Turkey

**Keywords:** *Aedes albopictus*, Geometric morphometry, Altitudinal transect, Albania

## Abstract

The rapid spread and settlement of *Aedes albopictus* mosquitoes across at least 28 countries in Europe, as well as several countries in Asia Minor, the Middle East and Africa, has made it one of the most invasive species of all time. Even though the biology of *Ae. albopictus* in its native tropical environment has been documented for a long time, the biology and ecology of this species in newly colonized temperate environments remain poorly known despite its important role as a vector for about twenty arboviruses. In this context, the main goals of this work were to investigate *Ae. albopictus* phenotypic variations at a local scale in Albania, the country where *Ae. albopictus* was first recorded in Europe, and to determine if its phenotypes could be affected by altitude. Analysis of *Ae. albopictus* wing phenotypes was performed using a geometric morphometric approach. We observed shape and size variations among altitudinal populations of *Ae. albopictus*. Differences of wing phenotypes were highlighted between altitude groups for male and female mosquitoes. The phenotypic variations observed in *Ae. albopictus* between altitudinal groups indicated these populations are exposed to environmental and ecological pressures. These results suggest the presence of phenotypic plasticity in this species.

## Abstract (fr)

La propagation et l’établissement rapides du moustique *Aedes albopictus* dans au moins 28 pays d’Europe, ainsi que dans plusieurs pays d’Asie mineure, du Moyen-Orient et d’Afrique, en ont fait l’une des espèces les plus invasives de tous les temps. Bien que la biologie d’*Ae. albopictus* dans son milieu tropical naturel soit documentée depuis longtemps, la biologie et l’écologie de cette espèce dans les milieux tempérés nouvellement colonisés restent mal connues malgré son rôle important de vecteur d’une vingtaine d’arbovirus. Dans ce contexte, les principaux objectifs de ce travail étaient d’étudier les variations phénotypiques d’*Ae. albopictus* à l’échelle locale en Albanie, le pays où *Ae. albopictus* a été signalé pour la première fois en Europe, et de déterminer si ses phénotypes pourraient être affectés par l’altitude. L’analyse des phénotypes des ailes d’*Ae. albopictus* a été effectuée en utilisant une approche de morphométrie géométrique. Nous avons observé des variations de forme et de taille parmi les populations altitudinales d’*Ae. albopictus*. Des différences de phénotypes d’ailes ont été mises en évidence entre les groupes d’altitude pour les moustiques mâles et femelles. Les variations phénotypiques observées chez *Ae. albopictus* entre les groupes altitudinaux indiquent que ces populations sont exposées à des pressions environnementales et écologiques. Ces résultats suggèrent la présence d’une plasticité phénotypique chez cette espèce.

## Introduction

The rapid spread and settlement of *Aedes* (*Stegomyia*) *albopictus* across at least 28 countries in Europe, as well as several other countries in Asia Minor, the Middle East and Africa, has made it one of the most invasive species of all time [9, 25]. This mosquito species is a potential vector for about twenty arboviruses, including zika, chikungunya and dengue [19]. The health challenges surrounding *Aedes albopictus* are therefore particularly important [21]. For example, in the past decade, its role in substantial epidemics of the chikungunya virus (CHIKV) in Italy [31] demonstrates the consequences of its ability to colonize and adapt to new environments, and especially water containers in urban sites.

*Aedes albopictus* is often described as a tropical mosquito species from South-East Asia but its true range extends to latitudes in the North-West of China. Indeed, this species can spend the winter in diapause and its eggs resist temperatures as low as −5 °C [23]. In Europe, this species was reported for the first time in 1979 in Albania [1], and it is now widespread and commonly found in this country, even at high altitudes (>1200 m) [42].

Nevertheless, even though the biology of *Ae. albopictus* in its native tropical environment has been documented since the 1980s [21], the biology and ecology of this species in newly colonized temperate environments remain poorly known despite its vectorial importance. These environments are characterized by greater climatic variations, which can impact the mosquitoes’ population dynamics.

Geometric morphometry gathers together a set of methods that allow the study of phenotypes and provides information on shape and size variations, and the relationships between these two variables [14]. The morphometric study of mosquitoes can highlight a correlation between the environment and the mosquito phenotypes [32]. For example, a previous study showed that *Ae. albopictus* populations from different geographical regions presented significant morphometric wing shape variations [12]. Moreover, adult mosquito size can be directly influenced by the environmental conditions they underwent during larval development [3]. Additionally, it can also be correlated with many life history traits such as fecundity [8] and longevity [47].

This is the first study in Albania using a geometric morphometric approach to investigate phenotypic variations of *Ae. albopictus* populations. The main goal of this study was to understand the possible phenotypic differences of *Ae. albopictus* populations on an altitudinal transect.

## Materials and methods

### Study area

**Figure 1:**
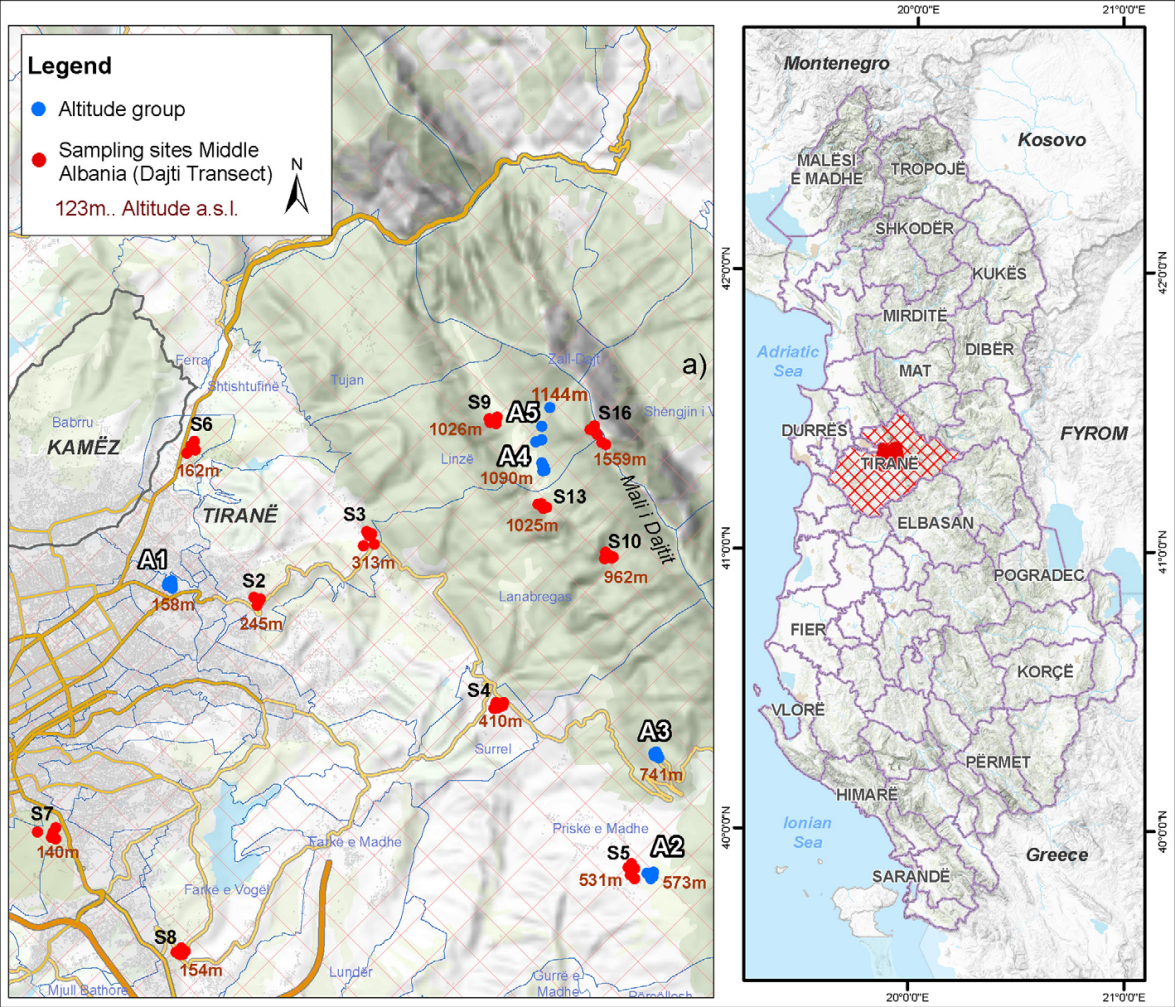
Map of the study area. Blue circles: altitude groups, red circles: sampling sites.

The study area was located on the Tirana-Dajti Mount in Albania (19°55′51.2″ E, 41°21′34.5″ N). This region is influenced by Mediterranean climate characterized by wet winters with a rainfall average of 1297 mm and average monthly temperatures of +24 °C in July and +6 °C in January. Eggs of *Ae. albopictus* were collected at 16 sites across a 154–1559 m altitude gradient transect (Fig. 1). In order to test the altitudinal effect on *Ae. albopictus* wing size and shape, five sites were selected along this transect and categorized into five altitude groups (A1–A5) (Table 1). The first altitude group A1 (158 m) is an artificial, non-agricultural and green urban area located in Tirana. The second altitude group A2 (595 m) is rural, agricultural, non-irrigated arable land. The third altitude group A3 (762 m) is a mixed forest and semi-natural area. The fourth and fifth altitude groups A4 (1099 m) and A5 (1140 m), respectively, are mixed forest and semi-natural areas located on the top of the Dajti Mount close to each other (Fig. 1).

**Table 1:** Description of the sampling stations in our study area and number of *Aedes albopictus* wings analyzed by station for the geometric morphometric analysis.

Altitude group	District	Coordinates		Altitude (m)	Biotope	Number of wings by gender		
		North	East			♀	♂	Total
A1	Tirana	41.34705	19.85103	158	Urban area	25	10	35
A2	Priske, Tirana	41.31072	19.93309	595	Rural area	5	20	25
A3	Dajt, Tirana	41.32607	19.93374	762	Forest	44	70	114
A4	Dajt, Tirana	41.36240	19.91402	1099	Forest	37	18	55
A5	Dajt, Tirana	41.36663	19.91371	1140	Forest	0	39	39
Total						111	157	268

♀, female; ♂, male.

### Mosquito collection, identification and larval rearing

*Ae. albopictus* eggs were collected weekly between May and November 2013 with ovitraps. These traps are black cylindrical vessels (height: 9 cm; diameter: 11 cm) with an overflow hole (7 cm from the base) internally lined with heavy-weight seed germination paper [43]. They contained ~300 mL of tap-water with no attractants. Our study did not involve protected or endangered species. No specific permits were needed for these sites and/or activities; nevertheless, the landowner’s permission was always requested before any studies were performed on their properties.

Eggs laid on germination paper were transported to the Entomology laboratory of the Institute of Public Health (Tirana, Albania). After counting, species were identified based on their size, color, surface sculpting and shape [48]. The eggs selected from the five altitude groups collected between July and October 2013 (Table 1) were hatched at the Hacettepe University VERG Lab and reared using the CAA (Centro Agricoltura Ambiente, Bologna, Italy) standard procedure for mosquito rearing [6, 29]. Each egg paper strip was put in a 400 mL glass jar, filled with 350 mL of deionized water and 1 mL of hatching solution (12.5 g nutrient broth and 2.5 g brewer’s yeast in 100 mL deionized water). They were put inside a climatic chamber set at 25 °C and 80% relative humidity overnight. First instar larvae were counted on the next two days after the eggs hatched, then were randomly picked from each altitude group and were transferred to 1.5 L of deionized water in 31 × 20 cm pans, and held in a climate chamber (26 °C; 60–70% relative humidity; light and dark cycle of 16 h and 8 h) to be reared to adult stage for wing geometric morphometric analysis. The rest of the larvae were terminated. Starting from the day they were picked, the larvae from each condition were given 0.5 mg/larva of diet daily until pupation. The liquid diet consisted of tuna meal (TM, 50% w:v), bovine liver powder (BLP, 36% w:v), and inactivated brewer’s yeast (BY, 14% w:v) with added multiple vitamins (VM, 0.2 mg VM by 100 mL of diet) [6]. Pupae were transferred to 200 mL plastic cups filled with deionized water. Finally, emerging adults were transferred individually into labelled 1.5 mL Eppendorf tubes containing 96% ethanol.

### Wing preparation

For the geometric morphometric analysis, a total of 268 *Ae. albopictus* wings were used (111 females and 157 males) (Table 1). Wings were shaved by friction in a 50% ethanol solution, fixed on microscopic labeled slides with Euparal and flattened under cover slips. The wing slides were photographed with a scale, then digitized, archived and analyzed.

### Morphometric analysis

**Figure 2:**
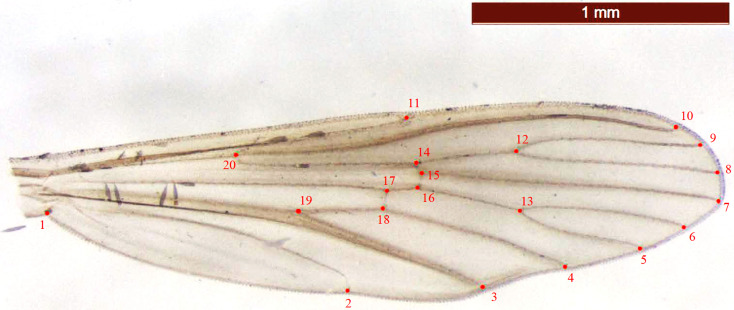
Example of an *Aedes albopictus* wing used for the geometric morphometric analysis. Red circles: landmarks.

First, we entered the scale pictures into tps-Util 1.60 [36]. For the analysis, we then used 20 landmarks [24] following the method of Rohlf and Slice [37] using tpsDIG2 2.18 software [35]. The intersections of wing veins with the wing margin, cross veins and major veins were used as locations for landmarks (Fig. 2). Morphometric analyses and graphical outputs were performed using various modules of the CLIC software [15]. Centroid sizes are described as the square root of the sum of squared distances of a set of landmarks from their centroid, i.e. the square root of the sum of the variances of the landmarks around that centroid in *x*- and *y*- directions [10]. They were used as a size estimator and compared with R 3.1.2 [41] using a Kruskal–Wallis test or nonparametric Wilcoxon–Mann–Whitney test followed by a post-hoc test (Mann–Whitney tests with Bonferroni correction). In order to calculate partial warps (shape variables), landmark configurations were scaled, translated, and rotated against the consensus configuration using the GLS Procrustes superimposition method [2, 10, 33, 34]. To compare population samples, we used the principal components (PC), which are based on the partial warps [10]. Pairwise distances of Mahalanobis between populations were calculated with CLIC [15] and tested by nonparametric permutation tests (1000 iterations) in order to evaluate the degree of similarity between populations. The percentage of correctly assigned individuals to the corresponding group was assessed by a simple reclassification test for each individual using Mahalanobis distances. These distances were then used to construct a UPGMA tree to examine the similarities among populations with PAST (version 3.25) [20]. The thin-plate spline was computed using the software MorphoJ (version 1.07) [22] in order to visualize the shape changes in the wings between the groups tested [49]. The contribution of size to wing shape (residual allometry) was estimated by multivariate regression of partial warps on size. Finally, the presence of isolation by distance was researched using a Mantel test (correlation between Procruste values and geographical distances in meter) under PAST (version 3.25).

Analyses were performed by altitude groups. It was not possible to test the effect of month on wing morphometry because of the small number of specimens captured each month.

## Results

### Sexual dimorphism

Distances of Mahalanobis were significantly different between both genders (Adjusted *p*-values < 0.0001) highlighting the wing shape variations between males and females (Supplementary Table S1). The observed differentiation between the two groups was supported by the simple reclassification scores (98% for females and 100% for males). Centroid sizes, used as the wing size measure, were significantly different between genders (Wilcoxon–Mann–Whitney Test: *p-*value < 0.0001), with males revealing smaller wings than females (Supplementary Fig. S1). The size contribution to wing shape differentiation was 61% (Supplementary Fig. S2).

Analyses were performed separately for males and females because phenotypic differences were found between sexes.

### Altitudinal differentiation for females

**Figure 3:**
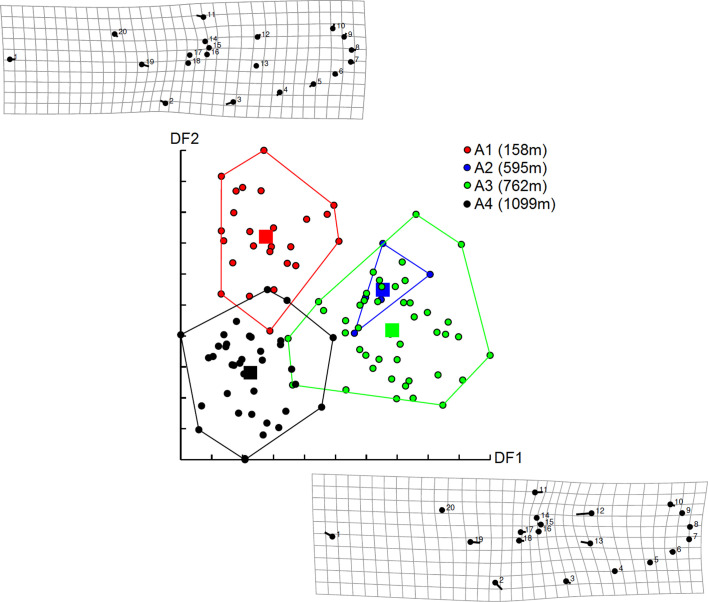
Distribution of *Aedes albopictus* females along the first two discriminant factors (DF) of shape analysis by altitude groups. This distribution was based on the partial warps. Horizontal axis: discriminant factor 1; Vertical axis: discriminant factor 2. Altitude groups: A1 (158 m), A2 (595 m), A3 (762 m), A4 (1099 m). Signs indicate each individual.

**Figure 4:**
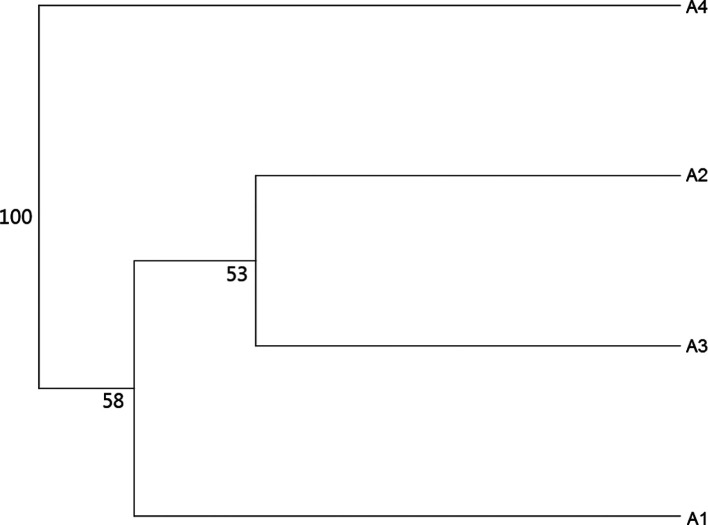
UPGMA tree for *Aedes albopictus* females based on Mahalanobis distances with 1000 bootstrap replicates. Altitude groups: A1 (158 m), A2 (595 m), A3 (762 m), A4 (1099 m).

As previously mentioned, the effect of altitude on *Ae. albopictus* phenotypes was tested separately by gender (see the sample size in Table 1). For females, wing shapes studied by distances of Mahalanobis were significantly different between all groups of altitude, except between groups A1 and A2 and between groups A2 and A3 (adjusted *p-*value < 0.00833, four components, 63.45% of total shape variance) (Fig. 3 and Supplementary Fig. S3). The observed differentiation between samples was supported by the simple reclassification scores with an average for altitudinal groups of 93.75% (88–100). The UPGMA tree showed similar results. Population A4 segregated into a single branch (100 bootstrap value, high dissimilarity) and other populations (A1–A3) were on the second branch (53 and 58 bootstrap value) (Fig. 4).

**Figure 5:**
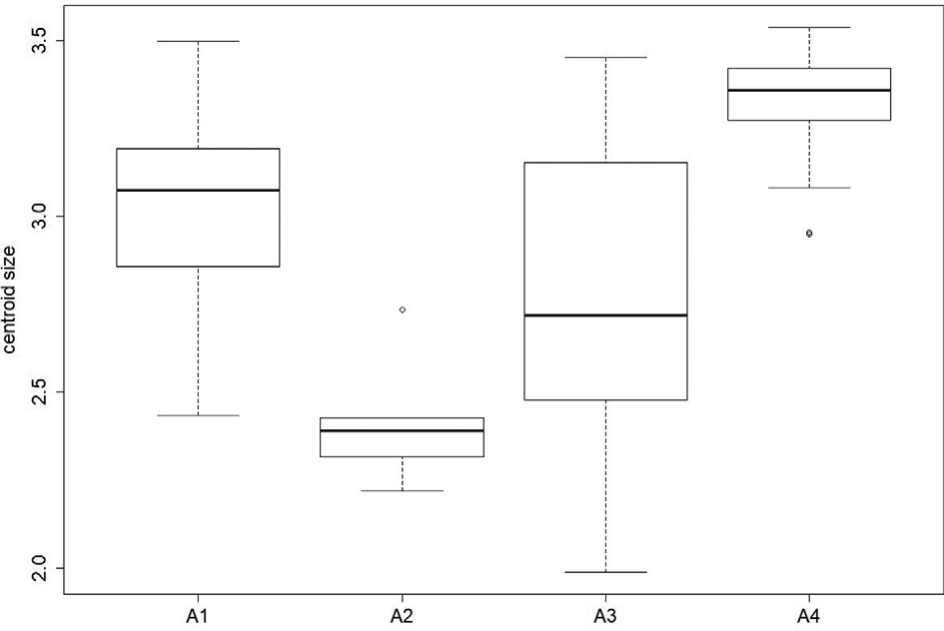
Boxplot of female centroid sizes by altitude groups. Altitude groups: A1 (158 m), A2 (595 m), A3 (762 m), A4 (1099 m).

**Figure 6:**
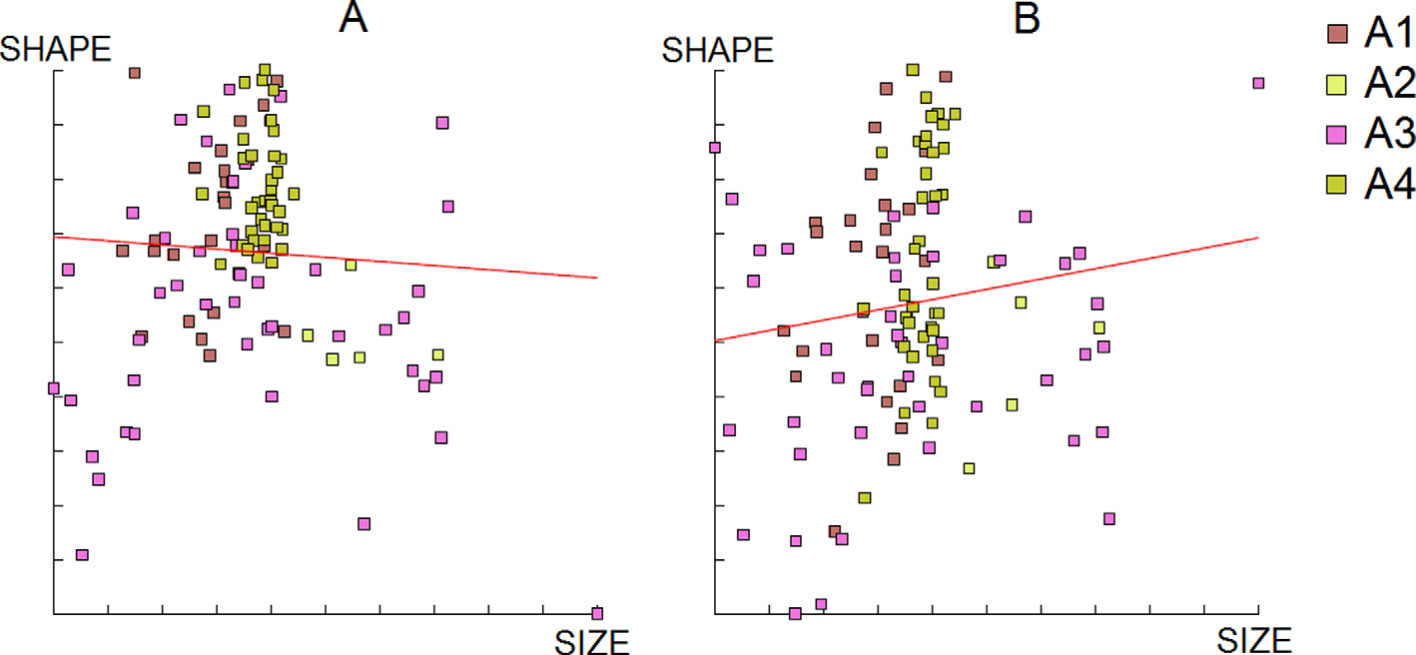
Regression of the first two discriminant factors (DF) of shape analysis on centroid size from *Aedes albopictus* females. Horizontal axis: centroid size of the wing; Vertical axis (A): discriminant factor 1, representing 24% of the total discrimination; Vertical axis (B): discriminant factors 2, representing 17% of the total discrimination. This regression was based on the partial warps. Regression line is shown. Squares indicate individual mosquitoes. Altitude groups: A1 (158 m), A2 (595 m), A3 (762 m), A4 (1099 m).

We also observed a significant altitudinal effect on wing size (centroid size) for females (*χ* ^2^ = 52.508, *p*-value < 0.0001). The post-hoc test highlighted significant differences between all groups except between A2 and A3 *(p-*value = 0.4007) (Fig. 5). The size contribution to wing shape differentiation was 0% and 1% (Fig. 6). Group A5 (1140 m) was not represented for females since no wings were available for this altitude (Table 1).

The Mantel test highlighted a weak and non-significant correlation between Procrustes values and geographical distance (*r* ^2^ = 0.16209, *p-*value = 0.3003).

### Altitudinal differentiation for males

**Figure 7:**
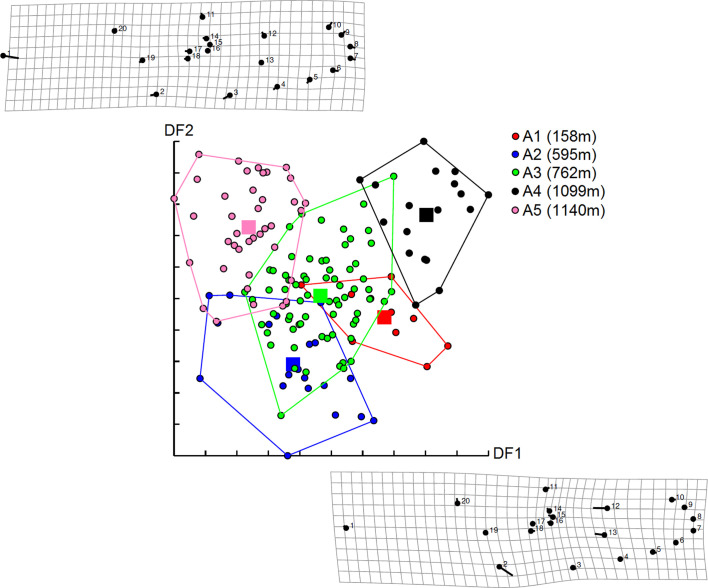
Distribution of *Aedes albopictus* males along the first two discriminant factors (DF) of shape analysis by altitude groups. This distribution was based on the partial warps. Horizontal axis: discriminant factor 1; Vertical axis: discriminant factor 2. Altitude groups: A1 (158 m), A2 (595 m), A3 (762 m), A4 (1099 m), A5 (1140 m).

**Figure 8:**
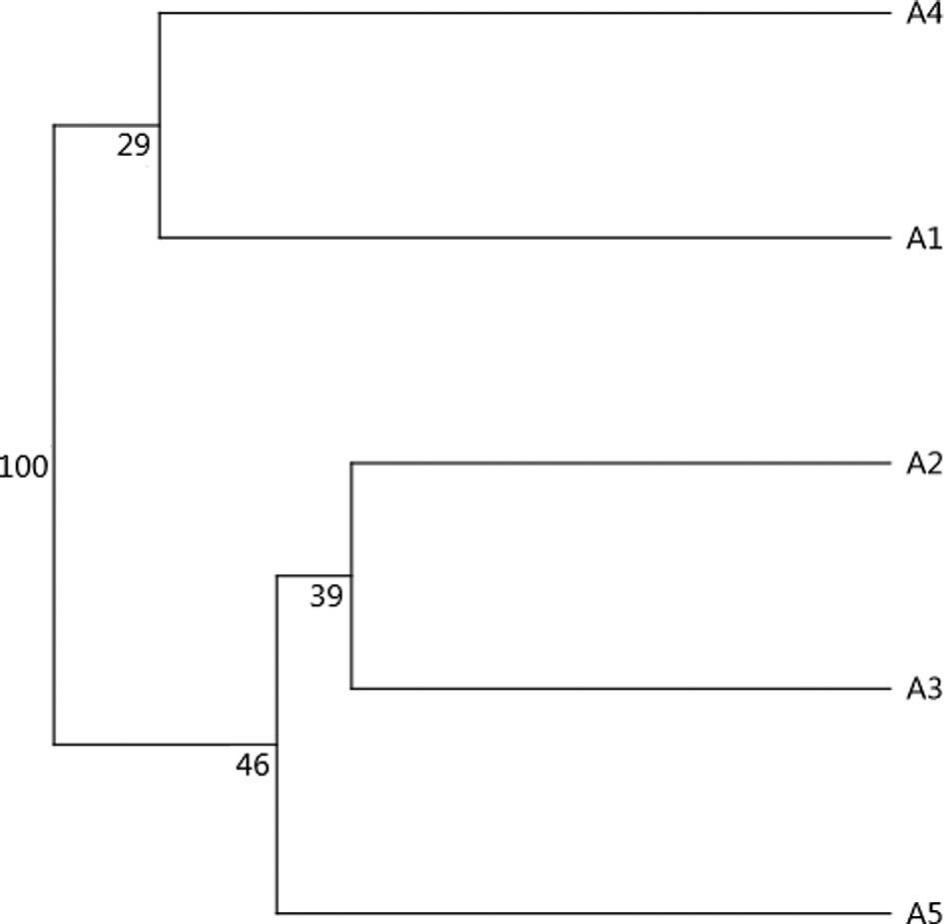
UPGMA tree for *Aedes albopictus* males based on Mahalanobis distances with 1000 bootstrap replicates. Altitude groups: A1 (158 m), A2 (595 m), A3 (762 m), A4 (1099 m), A5 (1140 m).

For males, Mahalanobis distances were significantly different between all altitudinal groups (Adjusted *p-*value < 0.005, nine components, 83.98% of total shape variance) (Fig. 7 and Supplementary Fig. S3). The observed differentiation between samples was supported by the UPGMA tree (Fig. 8) and the simple reclassification scores with an average of 89% for altitudinal groups (81–100%).

**Figure 9:**
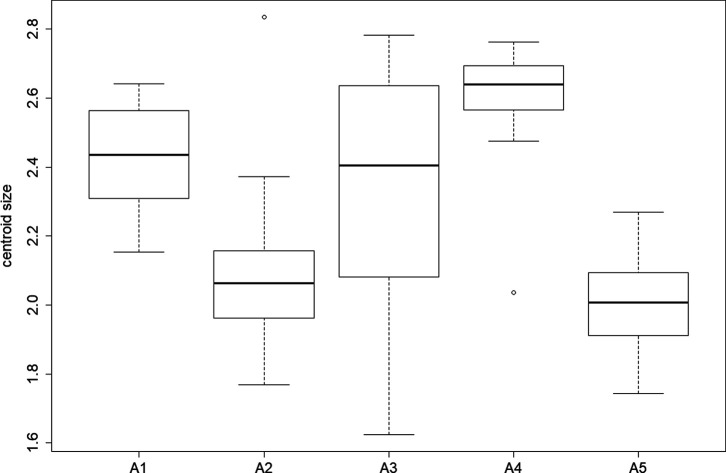
Boxplot of male centroid sizes by altitude groups. Altitude groups: A1 (158 m), A2 (595 m), A3 (762 m), A4 (1099 m), A5 (1140 m).

**Figure 10:**
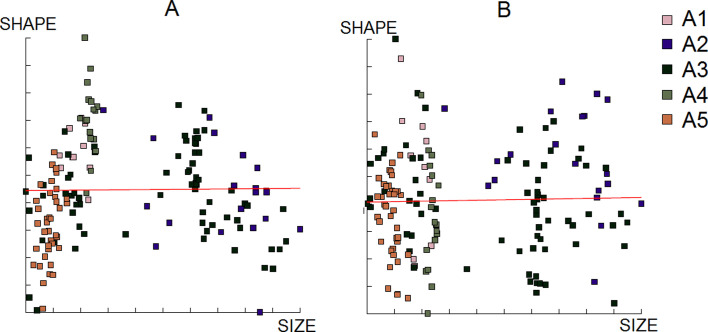
Regression of the first two discriminant factors (DF) of shape analysis on centroid size from *Aedes albopictus* males. Horizontal axis: centroid size of the wing; Vertical axis (A): discriminant factor 1, representing 19% of the total discrimination; Vertical axis (B): discriminant factors 2, representing 17% of the total discrimination. This regression was based on the partial warps. Regression line is shown. Squares indicate individual mosquitoes.

We also observed a significant altitudinal effect on wing size (centroid size) for males (*χ* ^2^ = 55.521, *p*-value < 0.0001). The post-hoc test highlighted significant differences between all groups, except A1 and A3 (*p-*value = 1.0000) and between A2 and A5 *(p-*value = 0.9805) (Fig. 9). The size contribution to wing shape differentiation was 0% and 0% (Fig. 10).

As for females, the Mantel test showed a weak and non-significant correlation between Procrustes values and geographical distance (*r* ^2^ = 0.09096, *p-*value = 0.7810).

## Discussion

This work is the first comparative geometric morphometric study of different *Ae. albopictus* populations in Albania. To begin with, geometric morphometric analyses on the wings of specimens captured along the transect showed sexual dimorphism between males and females, a common phenomenon in many insect species [7, 16, 44, 45]. Females present longer and more slender wings compared to males (Supplementary Table S1 and Fig. S1). This dimorphism can be classically explained by the different behaviors of males and females (blood meals, egg laying, light attractiveness, etc.). For example, males might need a smaller flying capacity in comparison with females since they do not require blood meals. This difference in behavior could explain the wing differences between genders. Further studies comparing dispersion capacity according to gender are necessary to confirm this hypothesis.

Second, differences in male and female wing phenotypes (shape and size) among groups were observed. Even though this study highlights wing differences between altitudinal groups, it was not possible to draw any conclusions regarding the effect of month on the mosquito phenotypes. In the case of small populations or a low number of captured individuals, sampling size is limiting for discriminant analyses. Indeed, the variance of the dataset cannot be explained due to the low number of principal components. Climatic and environmental conditions (temperature, relative humidity, variation between night and day, etc.) could exist depending on the month. Further studies should be considered with larger sample sizes according to month and altitude.

For males and females, the size and shape analysis indicated significant differences among altitudinal populations. This result is most probably associated with biological [18] and/or environmental factors such as different blood sources (different host populations available between stations), ecological factors like different microhabitats [27], ecosystem vegetation [13], and/or climatic effects such as temperature differences between altitudes groups [11, 26, 28].

Mosquito size can be directly influenced by environmental conditions undergone during larval development [3]. Individuals reared at higher temperatures are expected to have smaller wings [28, 30]. A previous study carried out in the same area already showed that altitude can be a proxy for temperature, which may impact the oviposition activity of *Ae. albopictus* [42]. Therefore, it is very likely that their egg stages have been subjected to different climatic conditions, generating phenotypic polymorphism in adults. It is known that climatic parameters influence the development of insect larvae and therefore the phenotype of the adult [3]. In particular, temperatures will greatly influence adult size, smaller adults emerge at high temperatures and larger adults at low temperatures [4]. Indeed, low temperatures will involve slow development and therefore large individuals. Our results highlight the presence of size differences between altitude groups with, at higher altitudes, females showing larger wings. Surprisingly, this association is not observed for the males from group A4 at 1099 m. These results associated with the presence of a sexual dimorphism reinforce the existence of gender-specific selection pressure. We also observed that group A1 (158 m) insects display larger wings than expected at low altitude. This station capture was located in the urban area of Tirana where the population of *Ae. albopictus* is more dense compared to higher altitudes. Larvae in the urban breeding sites might be under density and/or competition pressure or other factors related to city conditions. For example, a study showed that water containing more plant material accelerates *Ae. albopictus* larvae development [13]. The fact that less plant material might be present in urban breeding sites could also lead to longer development and therefore larger adults.

It is known that mosquito body size is correlated with numerous factors (ecological, environmental, physiological and genetic factors) [3, 38] and can be directly influenced by the environmental conditions undergone during larval development such as habitat quality [40], and larvae density and competition [17]. Since no information on the breeding sites was recorded (larvae density, water composition, etc.), we cannot predict or estimate how much these variables influence the size and shape variations. The possible correlations between geographic, climatic, genetic and phenotypic differentiations should be explored in specific and multidisciplinary studies based on larger sample sizes.

These selection pressures can also only be of environmental origin. A study [5] demonstrated for *Anopheles superpictus*, under laboratory conditions, the effect of temperature and humidity on wing deformation. These deformations can therefore also be explained by the variability of climatic conditions related to altitude and stations.

In addition, like many temperate species, *Ae. albopictus* uses diapause at the egg stage to survive the low temperatures in winter and delay reproduction until favorable climatic conditions arrive. As a key environmental signal, the shortening of days will cause females to lay diapause-programmed eggs (halted development, reduced metabolism, and increased resistance) [46]. It is therefore likely that climatic conditions, at the end and beginning of diapause, might also have played a role in the phenotypic differentiation observed in adults in our study. Further studies on detailed analysis of geometric morphometric data in comparison with temperature and relative humidity data are necessary to validate, or invalidate, this hypothesis.

A previous study showed that variability in *Ae. albopictus* wing size between separate populations was not associated with mating success [12]. Another study suggests that there might be heterogeneity in wing morphology within *Aedes aegypti* populations [39]. The shape and size differences between populations can be a result of phenotypic variability. Nevertheless, additional analyses are required to test for a correlation between genetic diversity and phenotypic diversity. This could reveal differences between *Ae. albopictus* populations and their ability to establish themselves in different environmental and climatic conditions at different altitudes.

To conclude, this study showed different types of phenotypic variations between local populations of *Ae. albopictus*: sexual dimorphism and phenotype variations (shape and size of wings) according to altitude for both genders. The phenotypic variation observed between these populations appears to be correlated to the local environmental and climatic variations between altitudinal groups. These results highlight the adaptability and plasticity of this mosquito species. Further studies are necessary in order to determine whether the populations were established in those environments or if they were introduced through passive transportation during summer.

## Conflict of interest

The authors confirm that there are no known conflicts of interest associated with this publication and there has been no significant financial support for this work that could have influenced its outcome.

## Author contributions

J.P., E.V. and F.G. carried out the experiments. J.P. analyzed the data. J.P., E.V., A.F. and A.M. wrote the manuscript with support from S.B and B.A. J.P., E.V., S.B. and B.A. conceived and planned the experiments. E.V., S.B., P.K. and K.M. contributed to field sampling, mosquito species identification and sample preparation.

## Supplementary Material

Supplementary material is available at https://www.parasite-journal.org/10.1051/parasite/2019053/olm
**Table S1.** Synthesis of the Mahalanobis distance results (and *p-*values). Values below the diagonal are Mahalanobis distances (and *p-*value, adjusted *p-*value < 0.00833) for *Aedes albopictus* females between altitude groups; values above the diagonal are Mahalanobis distances (and *p-*value, adjusted *p-*value < 0.005) for *Aedes albopictus* males between altitude groups; Altitude groups: A1 (158 m), A2 (595 m), A3 (762 m), A4 (1099 m), A5 (1140 m). **Fig. S1.** Distribution of *Aedes albopictus* individuals along the first discriminant factor (DF1) of shape analysis by genders. This distribution was based on the partial warps. Black bars: females; Gray bars: males. **Fig. S2.** Boxplot of centroid sizes for females and males. **Fig. S3.** First discriminant factor regression on centroid size. Vertical axis: discriminant factor 1, representing 100% of the total discrimination; Horizontal axis: centroid size of the wing. The analysis was based on the partial warps. White squares: females; Black squares: males. Regression line is shown.
