# Supplementary material for: Altitudinal variations in wing morphology of Aedes albopictus (Diptera, Culicidae) in Albania, the region where it was first recorded in Europe
Source: Parasite. 2019 Sep 6;26:55. doi: 10.1051/parasite/2019053 (PMC6729119; doi:10.1051/parasite/2019053)
Supplement: Supplementary file 1 — Table S1. Synthesis of the Mahalanobis distance results (and p-values). Values below the diagonal are Mahalanobis distances (and p-value, adjusted p-value < 0.00833) for Aedes albopictus females between altitude groups; values above the diagonal are Mahalanobis distances (and p-value, adjusted p-value < 0.005) for Aedes albopictus males between altitude groups; Altitude groups: A1 (158 m), A2 (595 m), A3 (762 m), A4 (1099 m), A5 (1140 m). [file parasite-26-55-s1.pdf]

Supplementary Material

Table S1. Synthesis of the Mahalanobis distances results (and *p*-value). Values below the diagonal are Mahalanobis distances (and *p*-value, adjusted *p*-value < 0.00833) for *Aedes albopictus* females between altitude groups; values above the diagonal are Mahalanobis distances (and *p*-value, adjusted *p*-value < 0.005) for *Aedes albopictus* males between altitude groups; Altitude groups: A1 (158 m), A2 (595 m), A3 (762 m), A4 (1099 m), A5 (1140 m).

		Altitude Group				
		A1	A2	A3	A4	A5
Altitude group	A1	-	3.97 (<0.0001)	3.33 (0.001)	3.88 (0.003)	4.68 (<0.0001)
	A2	4.90 (0.028)	-	2.68 (<0.0001)	4.98 (<0.0001)	3.53 (<0.0001)
	A3	3.97 (<0.0001)	3.87 (0.240)	-	3.72 (<0.0001)	2.74 (<0.0001)
	A4	3.25 (<0.0001)	5.16 (0.006)	3.86 (<0.0001)	-	4.89 (<0.0001)
	A5	/	/	/	/	-
