# Supplementary material for: Altitudinal variations in wing morphology of Aedes albopictus (Diptera, Culicidae) in Albania, the region where it was first recorded in Europe
Source: Parasite. 2019 Sep 6;26:55. doi: 10.1051/parasite/2019053 (PMC6729119; doi:10.1051/parasite/2019053)
Supplement: Supplementary file 2 — Fig. S1. Distribution of Aedes albopictus individuals along the first discriminant factor (DF1) of shape analysis by genders. This distribution was based on the partial warps. Black bars: females; Gray bars: males. [file parasite-26-55-s2.pdf]

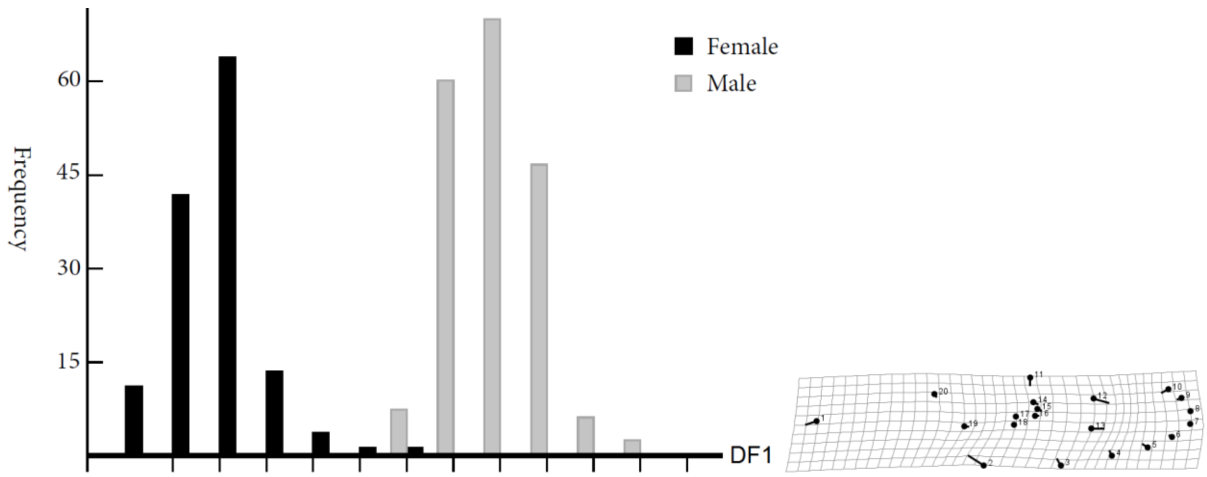

Fig. S1. Distribution of *Aedes albopictus* individuals along the first discriminant factor (DF1) of shape analysis by genders. This distribution was based on the partial warps. Black bars: females; Gray bars: males.
