# Supplementary figures and images for: Altitudinal variations in wing morphology of Aedes albopictus (Diptera, Culicidae) in Albania, the region where it was first recorded in Europe
Source: Parasite. 2019 Sep 6;26:55. doi: 10.1051/parasite/2019053 (PMC6729119; doi:10.1051/parasite/2019053)

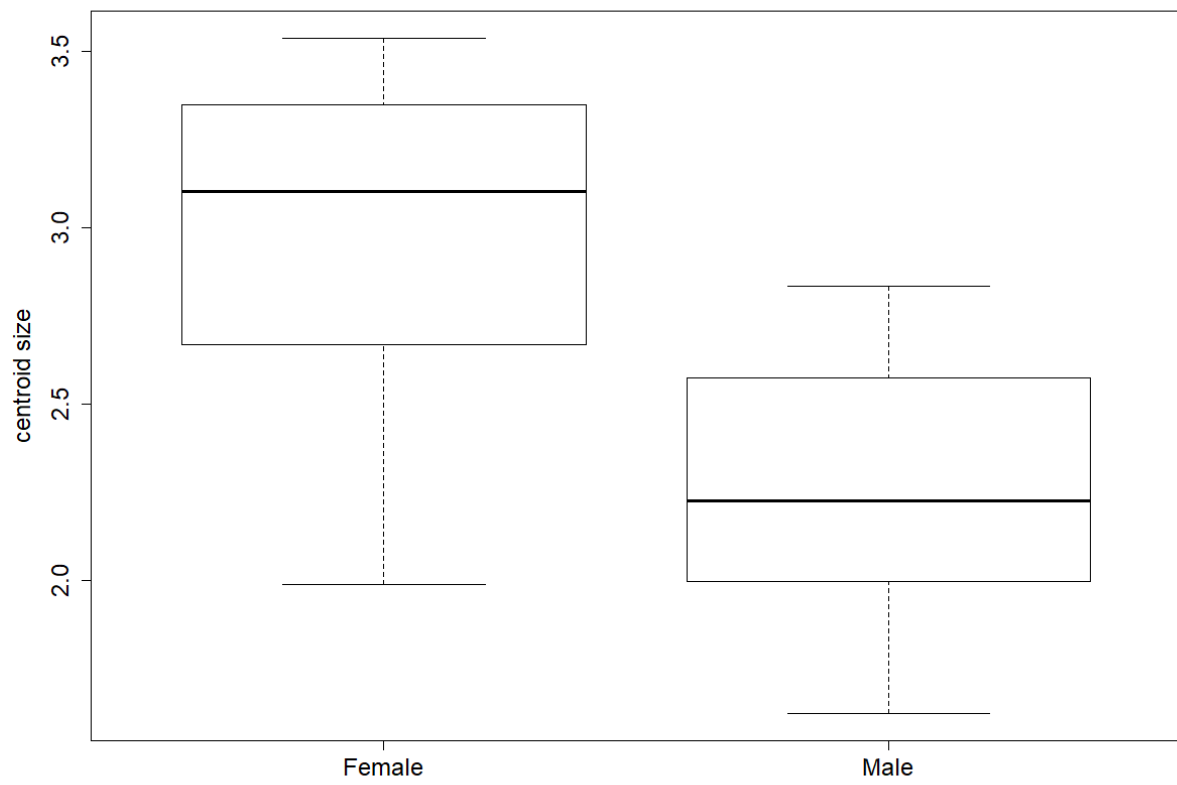

**Fig. S2.** Boxplot of centroid sizes for females and males.

Supplement: Supplementary file 3 — Fig. S2. Boxplot of centroid sizes for females and males. [file parasite-26-55-s3.pdf]
