# Supplementary material for: Altitudinal variations in wing morphology of Aedes albopictus (Diptera, Culicidae) in Albania, the region where it was first recorded in Europe
Source: Parasite. 2019 Sep 6;26:55. doi: 10.1051/parasite/2019053 (PMC6729119; doi:10.1051/parasite/2019053)
Supplement: Supplementary file 4 — Fig. S3. First discriminant factor regression on centroid size. Vertical axis: discriminant factor 1, representing 100% of the total discrimination; Horizontal axis: centroid size of the wing. The analysis was based on the partial warps. White squares: females; Black squares: males. Regression line is shown. [file parasite-26-55-s4.pdf]

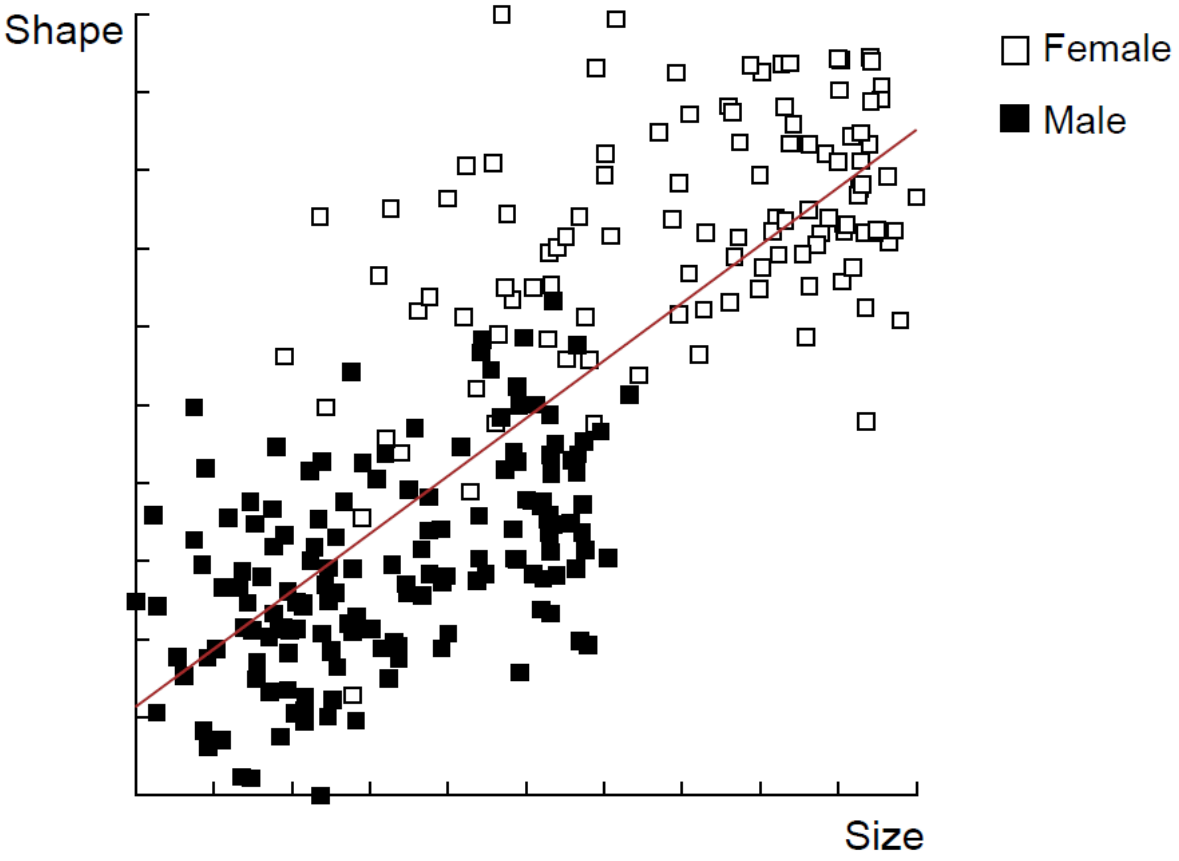

Fig. S3. First discriminant factor regression on centroid size. Vertical axis: discriminant factor 1, representing 100% of the total discrimination; Horizontal axis: centroid size of the wing. The analysis was based on the partial warps. White squares: females; Black squares: males. Regression line is shown.
